# Fine genetic mapping and transcriptomic analysis revealed major gene modulating the clear stripe margin pattern of watermelon peel

**DOI:** 10.3389/fpls.2024.1462141

**Published:** 2024-09-04

**Authors:** Shao Yang, Sikandar Amanullah, Yaru Duan, Yu Guo, Ming Xu, Xiuping Bao, Bohan An, Chengzhi Yuan, Xiujie Liu, Jixiu Liu, Yue Gao, Wen Zhao, Xinyuan Li, Meiling Gao

**Affiliations:** ^1^ College of Life Science, Agriculture and Forestry, Qiqihar University, Qiqihar, China; ^2^ Department of Horticultural Science, North Carolina State University, Mountain Horticultural Crops Research and Extension Center, Mills River, NC, United States; ^3^ Qiqihar Agricultural Technology Extension Center, Qiqihar, China; ^4^ Heilongjiang Provincial Key Laboratory of Resistance Gene Engineering and Protection of Biodiversity in Cold Areas, Qiqihar, China

**Keywords:** fruit peel stripe, fine genetic mapping, MYB transcription factor family, transcriptome analysis, watermelon

## Abstract

The peel stripe margin pattern is one of the most important quality traits of watermelon. In this study, two contrasted watermelon lines [slb line (P_1_) with a clear peel stripe margin pattern and GWAS-38 line (P_2_) with a blurred peel stripe margin pattern] were crossed, and biparental F_2_ mapping populations were developed. Genetic segregation analysis revealed that a single recessive gene is modulating the main-effect genetic locus (*Clcsm*) of the clear stripe margin pattern of peel. Bulked segregant analysis-based sequencing (BSA-Seq) and fine genetic mapping exposed the delimited *Clcsm* locus to a 19.686-kb interval on chromosome 6, and the *Cla97C06G126680* gene encoding the MYB transcription factor family was identified. The gene mutation analysis showed that two non-synonymous single-nucleotide polymorphism (nsSNP) sites [Chr6:28438793 (A-T) and Chr6:28438845 (A-C)] contribute to the clear peel stripe margin pattern, and quantitative real-time polymerase chain reaction (qRT-PCR) also showed a higher expression trend in the slb line than in the GWAS-38 line. Further, comparative transcriptomic analysis identified major differentially expressed genes (DEGs) in three developmental periods [4, 12, and 20 days after pollination (DAP)] of both parental lines. Gene ontology (GO) and Kyoto Encyclopedia of Genes and Genomes (KEGG) functional enrichment analyses indicated highly enriched DEGs involved in metabolic processes and catalytic activity. A total of 44 transcription factor families and candidate genes belonging to the ARR-B transcription factor family are believed to regulate the clear stripe margin trait of watermelon peel. The gene structure, sequence polymorphism, and expression trends depicted significant differences in the peel stripe margin pattern of both parental lines. The *ClMYB36* gene showed a higher expression trend for regulating the clear peel stripe margin of the slb line, and the *ClAPRR5* gene depicted a higher expression for modulating the blurred peel stripe margin in the GWAS-38 line. Overall, our fine genetic mapping and transcriptomic analysis revealed candidate genes differentiating the clear and blurred peel stripe patterns of watermelon fruit.

## Introduction

Watermelon (*Citrullus lanatus* L., 2n = 2x = 22) is a valuable fruit crop of the Cucurbitaceae family. It is primarily categorized into different sub-species of the main species (*C. lanatus*), e.g., 1) *C. lanatus* (Thunb.) Matsum. & Nakai, 2) *Citrullus amarus* Schrad., and 3) *Citrullus mucosospermus* Fursa (Fursa, 1972; Renner et al., 2014; Paris, 2015). It has become an excellent model plant for dissecting the essential biological pathways involved in the regulation of numerous complex traits. The considerable multiple breeding efforts made effective possibilities for the genetic improvement of watermelon (Pan et al., 2020). Among the world’s watermelon-producing countries, China ranks first with 60 million tons of production (Grumet et al., 2021).

The peel traits directly determine the commercial worth of watermelon fruit. The visual peel stripe margin pattern is supposed to be a naturally occurring phenomenon and can usually be seen in the longitudinal direction over the outer surface of the fruit (Zhai et al., 2019). Watermelon peel stripes exhibit different types of genetic and visual patterns as compared with the fruits of other Cucurbitaceae crops, e.g., pencil stripes, intermittent stripes, continuous stripes, reticular stripes, yellow belly stripes, spotted stripes, clear stripes, and blurred stripes; however, light green color stripes and mixed dark green color stripes are commonly found in different cultivars of watermelon (Gusmini and Wehner, 2006; Yang et al., 2015; Maragal et al., 2019; Liang et al., 2022; Amanullah et al., 2023).

The differentiation in peel colors takes place due to the accumulation of various pigment substances in peel tissues (Li et al., 2013; Wang et al., 2017). A total of three alleles for the watermelon peel stripes color have been proposed, e.g., dark green (*G*), light green (*gs*), and gray (*g*), and this allelic nature showed that *G* is dominant in both *gs* and *g*, but *gs* is dominant in *g* (Eyberg et al., 1980; Porter, 2013). Genetic analysis also disclosed that a dominant *ins* gene controls the spotted stripes and that the *gs* gene controls the light green stripes (Gusmini and Wehner, 2006); however, yellow color stripes have dominance over green color stripes (Yang et al., 2015). Further, the genetic model of three loci positioned on different chromosomes (4, 6, and 8) controlling different stripe patterns was also proposed, e.g., external stripe pattern (*S*), internal peel color (*Dgo*), and peel color depth (*D*) (Park et al., 2016). Some molecular mapping studies have identified a stable major-effect genetic region located on chromosome 8 (Chr-08), controlling the fruit peel color of watermelon (Park et al., 2016; Zhang et al., 2018; Li et al., 2019); however, a major gene (*ClCG08G017810* “*ClCGMenG*”, encoding 2-phytyl-1,4-beta-naphthoquinone methyltransferase) was significantly identified (Li et al., 2019). The multi-allelic *APRR2* gene and transcription factor were also found to be common causatives for the pigment accumulation and qualitative difference in the dark and light green peels of melon and watermelon (Oren et al., 2019).

Some studies have proposed the main-effect locus (*g*) that harbors major genes and alleles modulating the differentiated appearance of peel stripes of watermelon, e.g., medium to solid dark green (*G*), medium type (*gM*), wide type (*gW*), gray (*g*), and narrow type (*gN*) (Lou and Wehner, 2016). Two flanking markers (MCPI_05 and MCPI_16) located on chromosome 6 showed a positive and significant allele-specific contribution to the genetic inheritance of the striped peel trait in watermelon (Gama et al., 2015). A single dominant gene (*ClSP*) was spotted in the genetic segment (611.78 kb) of Chr-06, signifying the regulation of dark green stripes (Yue et al., 2021). Quantitative trait locus (QTL) positioned on Chr-09 also pinpointed two major genes (*Cla97C09G175150* and *Cla97C09G175170*) involved in the modulation of the dark green stripes and inter-stripe color of watermelon peel (Maragal et al., 2022). The genetic segment (62.50 kb) on Chr-06 exposed four potential genes (*Cla97C06G126560*, *Cla97C06G126680*, *Cla97C06G126710*, and *Cla97C06G126770*) regulating the differentiated watermelon stripes (Guo et al., 2019; Hou et al., 2022; Liang et al., 2022). A major-effect locus (*ClGS*) was identified in the genetic interval (107 kb) over Chr-06, and the candidate gene (*Cla019205*) controlling the dark green stripes of watermelon was predicted (Wang et al., 2022). Fine genetic mapping identified the main *ClIS* locus (160-kb region) on chromosome 6, and a single major gene (*Cla019202*) controlling intermittent stripe over watermelon peel was identified (Wang et al., 2023). It was also proposed that the inheritance of clear and blurred peel stripes is differentiated by the single recessive gene (*csm*), and the stripe width is related to the stripe type. However, blurred peel stripes only exist in fruits with medium and wide stripes; clear peel stripes are only observed in fruits with narrow stripes (Lou and Wehner, 2016).

In recent decades, advanced omics approaches and publicly available reference genome assemblies of watermelon (Guo et al., 2013, 2019; Wu et al., 2019) effectively encouraged molecular genetics and breeding studies. The bulked segregant analysis-based sequencing (BSA-Seq) approach assisted in rapid detection and fine mapping of the main-effect genetic locus harboring the candidate functional genes governing several differentiated phenotypes (Li et al., 2011; Li et al., 2017; Liu et al., 2019; Luan et al., 2019; Sun et al., 2020; Pei et al., 2021; Yang et al., 2021; Zhang et al., 2021; Lv et al., 2022a; Liang et al., 2022; Mi et al., 2023; Wang et al., 2023). Further, RNA sequencing (RNA-Seq), known as transcriptomic approach, has been widely applied for the identification of differentially expressed genes (DEGs) involved at the transcriptional and translational levels of the regulatory pathways of many significant traits of melon and watermelon (Zhu et al., 2017; Yang et al., 2022; Lv et al., 2022a; Zhan et al., 2023).

To date, there has been a significant focus on exploring the watermelon peel traits. Although some key genes have been pinpointed in many molecular studies, in-depth genetic understanding is still needed in many unexplored botanical groups. Hence, in this study, we performed whole-genome BSA-Seq and RNA-Seq analyses for fine genetic mapping and detection of key genes involved in differentiating the fruit peel stripe margin trait in the developed biparental segregated mapping populations of watermelon, respectively.

## Materials and methods

### Development of mapping population

**Figure 1:**
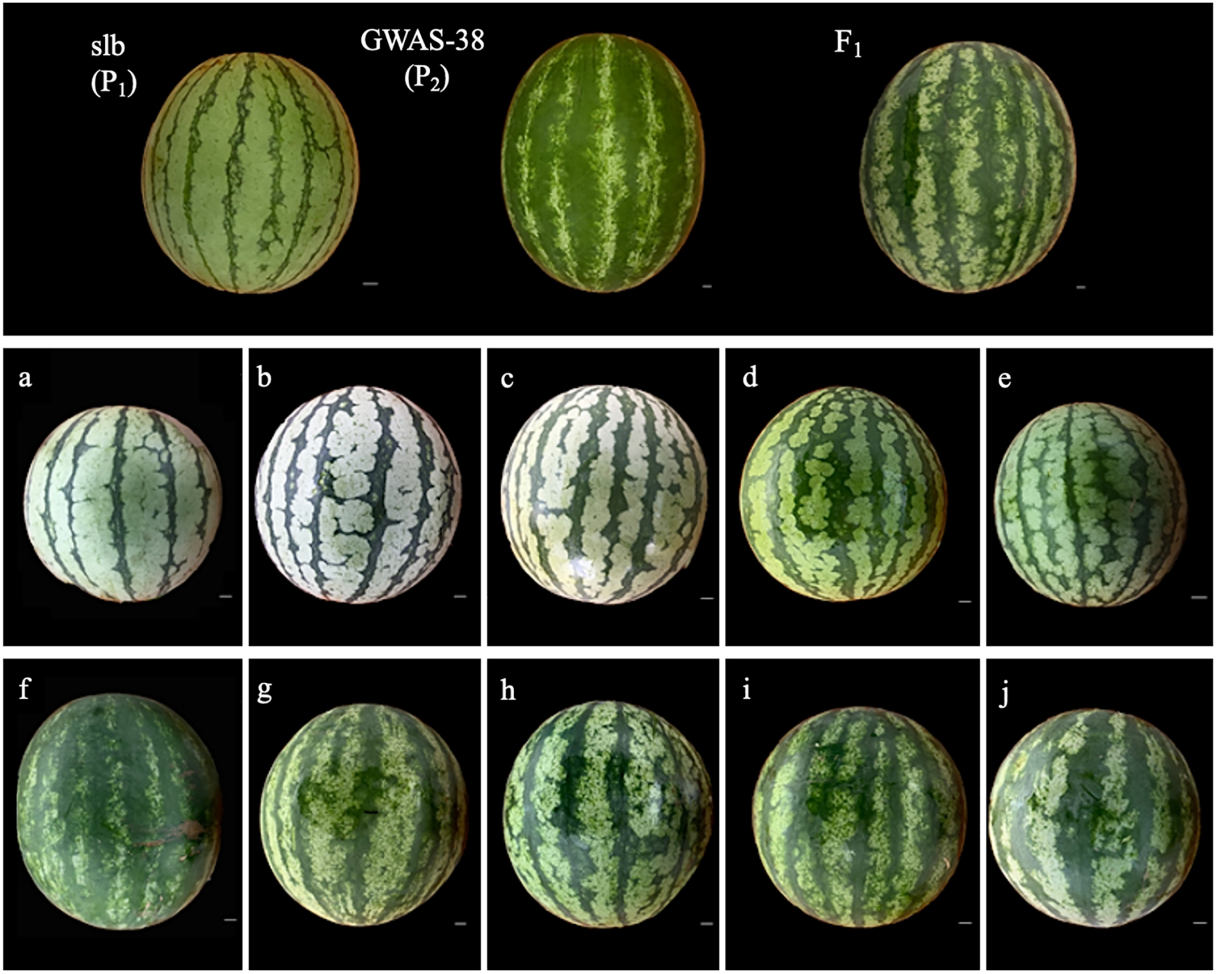
The comparative fruit peel stripe margin appearance of parental lines, F_1_, and F_2_ mapping populations. **(A–E)** Observations of clear peel stripe margin in fruits of F_2_ population. **(F–J)** Observations of blurred peel stripe margin in fruits of F_2_ population. Scale, 1 cm.

Two different types of watermelon parent materials, slb (P_1_, female parental line with clear and narrow stripe margin on fruit peel surface) and GWAS-38 (P_2_, male parental line with blurred and wide stripe margin on fruit peel surface), were selected from the natural germplasm resources of 144 accessions of genome-wide association study (GWAS) and used as experiment materials. The plant cultivation was conducted at the Qiqihar Agricultural Technology Extension Center, Qiqihar, P.R. China. The selected contrasted parental lines (slb and GWAS-38) were crossed to develop the F_1_ hybrid and biparental F_2_ mapping populations, respectively. The primary differences in the peel stripe margin pattern on the watermelon fruit peel of parental lines and developed mapping populations can be seen in **Figure 1**.

In the first experimental year of 2020, a total of 20 plants of P_1_ (GWAS-38), P_2_ (slb), and F_1_ hybrid were grown, and a primary F_2_ mapping population (*n* = 299 plants) was developed, which was used for the primary localization of the major-effect genetic loci (*Clcsm*) controlling clear stripe margins of watermelon peel. In 2021 and 2022, two extended F_2_ mapping populations (*n* = 613 plants and *n* = 1,454 plants) were used for fine mapping of the target locus and underlying genes. In addition, the plants of the natural population of 144 watermelon accessions (GWAS panel, widely collected from China and other countries around the world) were also grown and used for comparative experimental evaluation, respectively.

All the mapping populations were cultivated on the raised bed ridges in a plastic greenhouse by adopting the planting geometry of randomized complete block design (RCBD). A well-mixed soil with 10% potting mixture, 30% compost, and 60% loamy soil was used, and the plants were grown by retaining the distances of the plant to plant (70 cm) and row to row (60 cm) on bed ridges. All the necessary and regular horticultural practices were adopted for the raising of healthy crop plants.

### Phenotyping of peel stripe margin

The flowers of each plant population were manually pollinated at the anthesis period, and a single fruit from each plant was harvested after observing the suitable maturity indices at days after pollination (DAP). The freshly harvested fruits were photographed, and phenotyping of peel stripe margin patterns was performed on the basis of visual observation of a clear stripe margin pattern or a blurred stripe margin pattern over the peel surface of each fruit. The collected phenotypic dataset was scored as follows: 0 for clear stripe margin and 1 for blurred stripe margin. The final dataset was analyzed by performing the genetic segregation analysis using the chi-square (*χ* ^2^) test statistics of the IBM SPSS software (v25.0).

### DNA extraction and BSA sequencing

The juvenile leaves were sampled from each plant population, merged in liquid nitrogen, and cryopreserved at −8°C. Total genomic DNA was extracted using a modified protocol of cetyl trimethyl ammonium bromide (CTAB)-based extraction buffer (Allen et al., 2006), quantified with UV-Vis Spectrophotometer (DS-11FX, DeNovix, Wilmington, DE, USA), and stored at −20°C. Two DNA bulks from different peel stripe samples (20 clear peel stripes and 20 blurred peel stripes) were selected from primary F_2_ mapping populations (*n* = 299) and parental lines, and BSA-Seq was performed at a biotechnological company.

Whole-genome re-sequencing of comparative parental lines was performed at Beijing Genomics Institute (BGI), Shenzhen, China. The high-quality reads were filtered across the improved reference genome of watermelon (97103, v2) (http://www.cucurbitgenomics.org/) using the Burrows-Wheeler Aligner (BWA) software (Li and Durbin, 2009), and single-nucleotide polymorphism (SNP) variants were called using samtools and bcftools (Li et al., 2009; Danecek et al., 2021). The *delta* (Δ) single-nucleotide polymorphism (ΔSNP) index was computed as defined in a previous study (Li et al., 2017), where a ΔSNP index of 1 or 0 indicates a strong or no correlation between SNPs and the associated target trait, respectively. The significant association was statistically tested at *p* ≤ 0.01 using locally estimated scatterplot smoothing (LOESS) regression. Finally, a mapped region was considered a main-effect locus detected above the threshold level (Zhang et al., 2021).

### Development of genetic markers

First, the major-effect genetic locus was identified across the watermelon genome based on BSA-Seq analysis. Then, the cleaved amplified polymorphic sequence (CAPS) and insertion–deletion (InDel)-based markers were developed within the targeted locus using the filtered whole-genome sequenced reads of both parental lines. For the CAPS markers, the flanking genomic SNP locus sequences in the 500 base pairs (bp) were chosen and converted into markers using the SNP2CAPS software (Thiel et al., 2004; Amanullah et al., 2022). The InDel markers were developed based on the recently reported method and usage for molecular mapping (Mi et al., 2023). All the corresponding primer sequences were generated using the Primer Premier software (v6.25) (https://www.premierbiosoft.com/primerdesign/). The generated markers were synthesized and labeled with short abbreviations for ease of molecular experimentation of genotyping in biparental mapping populations, and information on forward and reverse primers, physical positions, product lengths, and endonucleases can be seen in **Supplementary Table S1**.

### Primary and fine genetic mapping

For the primary genetic mapping, the developed CAPS and InDel markers were checked within the three testing genotypes (P_1_, P_2_, and F_1_ hybrid), and a total of 19 polymorphic markers were used for genotyping within the primary F_2_ mapping population (*n* = 299). Fine genetic mapping was performed by narrowing down the primary mapped region of the *Clcsm* locus. The additional markers were developed and genotyped using the two expanded F_2_ mapping populations (*n* = 613 and *n* = 1,454), which helped in delimiting the maximum coverage of markers and exposed the final possible shortened region in kb interval, as well as screening of recombinant lines.

The optimized polymerase chain reaction (PCR) and the endonuclease digestion reactions for CAPS markers were performed as reported earlier (Amanullah et al., 2022). In short, the PCR amplification was performed using an Applied Biosystems 2720 Thermal Cycler (Hercules, CA, USA), and optimal reaction conditions were as follows: 1 μL of each DNA template, 1 μL of forward and reverse primers, 94°C for 5 min, followed by 35 cycles of 94°C for 1 min, with annealing temperature for 45 sec, and 72°C for 1 min. Then, the final PCR products were subjected to endonuclease digestion reactions using the mentioned temperature. For the association analysis, two InDel markers (TWIndel-3 and TWIndel-15) were used, and digested DNA products were electrophoresed in a 1% agarose gel. Then, the respective coded genotypic bands and phenotypic datasets were used for constructing the primary linkage map using the JionMap software (v4.0) (Ooijen, 2006).

### Candidate gene prediction, cloning, and association analysis

The candidate genes were predicted within the final delimited genetic interval, and the functional annotation of the identified gene was checked using the online databases of the watermelon genome (97103, v2, http://www.cucurbitgenomics.org/) and the National Center for Biotechnology Information (NCBI; https://www.ncbi.nlm.nih.gov/). Gene SNP mutation analysis was performed by checking the pairwise sequence of both parental lines (slb and GWAS-38) using the DNAMAN software (v10.0). The full-length sequence of the major *Clcsm* gene was retrieved from similar genomic information, gene primers were generated, and the major gene was cloned correspondingly. The information on forward and reverse sequences of functional genes and primers used for qRT-PCR and cloning can be seen in **Supplementary Table S2**.

In order to investigate the phylogenetic association of the Clcsm protein, the UniProt (https://www.uniprot.org) database was used to query the homologous amino acid sequences of candidate genes known in 13 species. The alignment of comparative protein sequences was conducted using the ClustalW software, and a phylogenetic tree was constructed based on bootstrap replications (1,000×) and a maximum likelihood approach using the MEGA software (v5.0, http://www.megasoftware.net/) (Felsenstein, 1981). The possible conserved domains were also detected by searching the web database of NCBI (https://www.ncbi.nlm.nih.gov/cdd).

### RNA extraction, RNA-Seq, and gene expression validation

The fruit peel stripes were regularly observed, and required stripe samples were collected at different developmental stages (4, 12, and 20 DAP) from both parental lines (slb and GWAS-38). Total RNA was isolated using the TRIzol reagent method (Rio et al., 2010), and cDNA was synthesized using the SureScript™ First-Strand cDNA Synthesis Kit by following the manufacturer’s guidelines. The expression of the candidate *Clcsm* gene was performed using the Step One Plus Real-Time qPCR platform (Applied Biosystems, Foster City, CA, USA). Gene-specific primers were designed based on the open reading frame sequence, an annealing temperature of 60°C, and an amplification length between 200 and 300 bp using the Primer Premier software (v6.25). The relative expression level of the target gene was calculated using the 2^−ΔΔCT^ technique (Livak and Schmittgen, 2001). qPCRs were conducted in a total of 10 µL of the reaction mixture (SYBR Green qPCR Master Mix) containing 0.02 µL of cDNA and 0.3 µL of each gene primer. A total of three technical replicates and three biological replicates of each fruit peel stripe sample were used for the relative gene expression analysis. The *β-Actin* gene was used as a reference gene to normalize the transcription levels (Kong et al., 2014).

For the transcriptomic analysis, RNA was similarly extracted from the collected fruit peel stripe sample groups at different developmental stages (4, 12, and 20 DAP) from both parental lines. RNA sequencing libraries were developed with 4 µg of isolated RNA using the Illumina TruSeq RNA sample preparation kit (FC-122-1001). After PCR amplification, RNA-Seq libraries of 300 bp were checked on a 2% ultra-agarose gel. The quality was checked by assessing the RNA integrated number (RIN > 8) and size of the library on a Bioanalyzer (2100, Agilent Technologies, Santa Clara, CA, USA). The good-quality RNA of peel stripe samples was placed on a Drikold and sent to Guangzhou Kidio Biotechnology Co., Ltd., for RNA sequencing and further analysis. The cDNA library was sequenced using Illumina NovaSeq 6000 from Gene Denovo Biotechnology Company (Guangzhou, China). The quality of the raw sequenced reads was estimated using FastQC (v0.11.2), all filtered clean-end reads were mapped across the watermelon reference genome (97103, v2) using TopHat (v2.0.11), and the final mapped reads of each transcript were calculated using HTSeq (v0.6.1). The raw RNA-Seq reads were uploaded to the Sequence Read Archive (SRA) database (PRJNA1070357, BioSample: SAMN39643078, *C. lanatus* transcriptome, and 18 independent libraries containing biological replicates) (https://www.ncbi.nlm.nih.gov/search/all/?term=PRJNA1070357) for the feasibility of the scientific community.

The candidate DEGs were screened and analyzed using the earlier reported methods (Yang et al., 2022; Lv et al., 2022b). The DEGs across the contrasted groups (G1, G2, and G3) were obtained based on the calculation of gene abundances and normalized reads per kb per million (RPKM) using the edgeR package (v4.0), and the screened DEGs were used for gene ontology (GO) and Kyoto Encyclopedia of Genes and Genomes (KEGG) enrichment analyses. The primers for candidate genes were designed, the relative expression levels of key genes were validated using qRT-PCR analysis, and significant differences were observed. The statistical differences in the expression trends between clear stripe margin and blurred stripe margin fruits were verified through statistical analysis (Student’s t-test) using the IBM SPSS software (v25.0). All the exported and synthesized primer pairs used for the experimental analysis of qRT-PCR can be seen in **Supplementary Table S4**.

## Results

### Analysis of genetic segregation of peel stripe margin

According to visual phenotypic observations, all the mature fruits of the slb line (P_1_) showed a clear peel stripe margin, and the GWAS-38 line (P_2_) exhibited a blurred peel stripe margin. All the fruits of F_1_ progeny exhibited the blurred peel stripe margin, consistent with the male parental line (GWAS-38, P_2_); however, the fruits of the F_2_ mapping population showed obvious variation, as shown in **Figure 1**. Among the harvested fruits of the primary F_2_ segregated population (*n* = 299), a total of 217 fruits with a blurred stripe margin pattern and 82 fruits with a clear stripe margin pattern over the peel surface of watermelon fruits were observed (**Table 1**), fitting a perfect 3:1 Mendelian ratio (*χ* ^2^ = 0.57, *p* > 0.05), which inferred that the clear stripe margin phenotype is controlled by a single recessive gene named *Clcsm* (a short abbreviation of *C. lanatus* L. and clear stripe margin). Further, among the fruit peel phenotypes and whole-genome sequencing data of the GWAS accession panel, the fruits of 116 watermelon lines depicted a clear peel stripe margin, and the fruits of 25 lines exhibited a blurred peel stripe margin surface (**Supplementary Table S3**).

**Table 1:** Genetic segregation analysis of peel stripe margin pattern in parental lines and derived mapping populations of watermelon.

Populations	Blurred stripe pattern	Clear stripe pattern	Segregation ratio	*χ* ^2a^	*p*-Value^b^
slb (P_1_)	0	20	-	–	–
GWAS-38 (P_2_)	20	0	-	–	–
F_1_	20	0	-	–	–
F_2_	217	82	3:1	0.57	0.45

a χ^2^ > χ^20.05^ = 3.841, indicating a significant difference.

b p < 0.05, indicating a significant difference.

### Analysis of primary mapping of *Clcsm* locus

**Figure 2:**
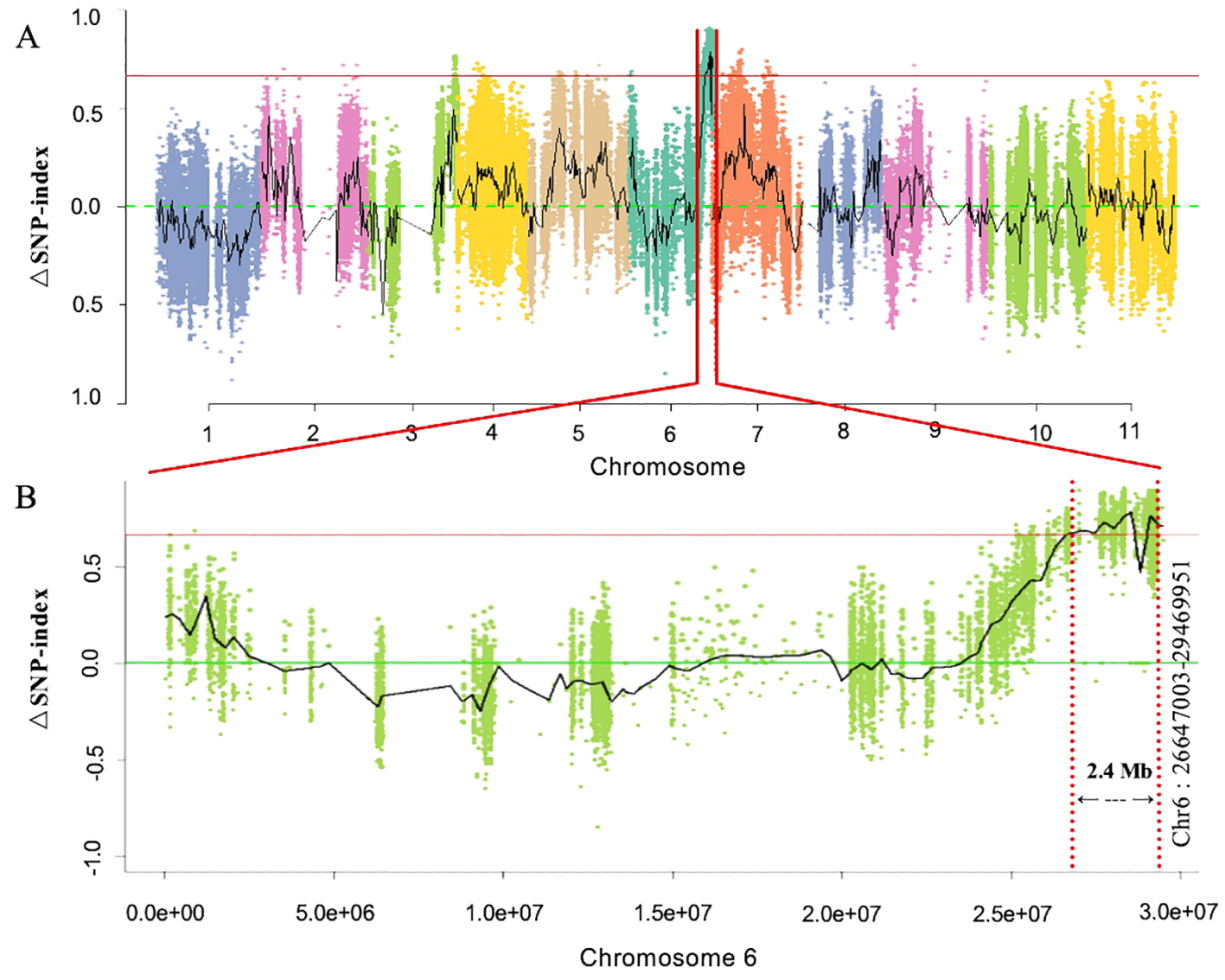
Identification of main-effect genetic locus controlling clear peel stripe margin of watermelon fruit based on BSA-Seq. **(A)** ΔSNP-index scan across whole-genome chromosomes. **(B)** ΔSNP-index scan across chromosome 6. Horizontal red lines denote the maximum threshold of 0.651 (*p* < 0.01). BSA-Seq, bulked segregant analysis-based sequencing; SNP, single-nucleotide polymorphism.

First, whole-genome BSA-Seq exposed the major-effect *Clcsm* locus positioned at the bottom end position of chromosome 6. A total of 87 and 83 million clean-end reads were obtained from both sequenced bulks (clear and blurred peel stripes); then, the low-quality sequencing reads were trimmed from the raw sequenced data, and filtered clean-end reads were retrieved. The final clean-end reads were multiply aligned and mapped onto the reference genome of watermelon (97103, v2). The whole-genome SNP index was calculated using the resequencing data of the gene pool with clear and blurred peel stripe margins of watermelon. The maximum threshold level of ΔSNP index was detected at 0.651 (*p* < 0.01), and a candidate genetic segment was detected on the bottom end position over chromosome 6 of the watermelon genome (**Figure 2A**). Meanwhile, the identified *Clcsm* gene locus was primarily located within the genetic segment (2.40 Mb, Chr6:26647003-29469951) (**Figure 2B**).

### Analysis of fine genetic mapping

**Figure 3:**
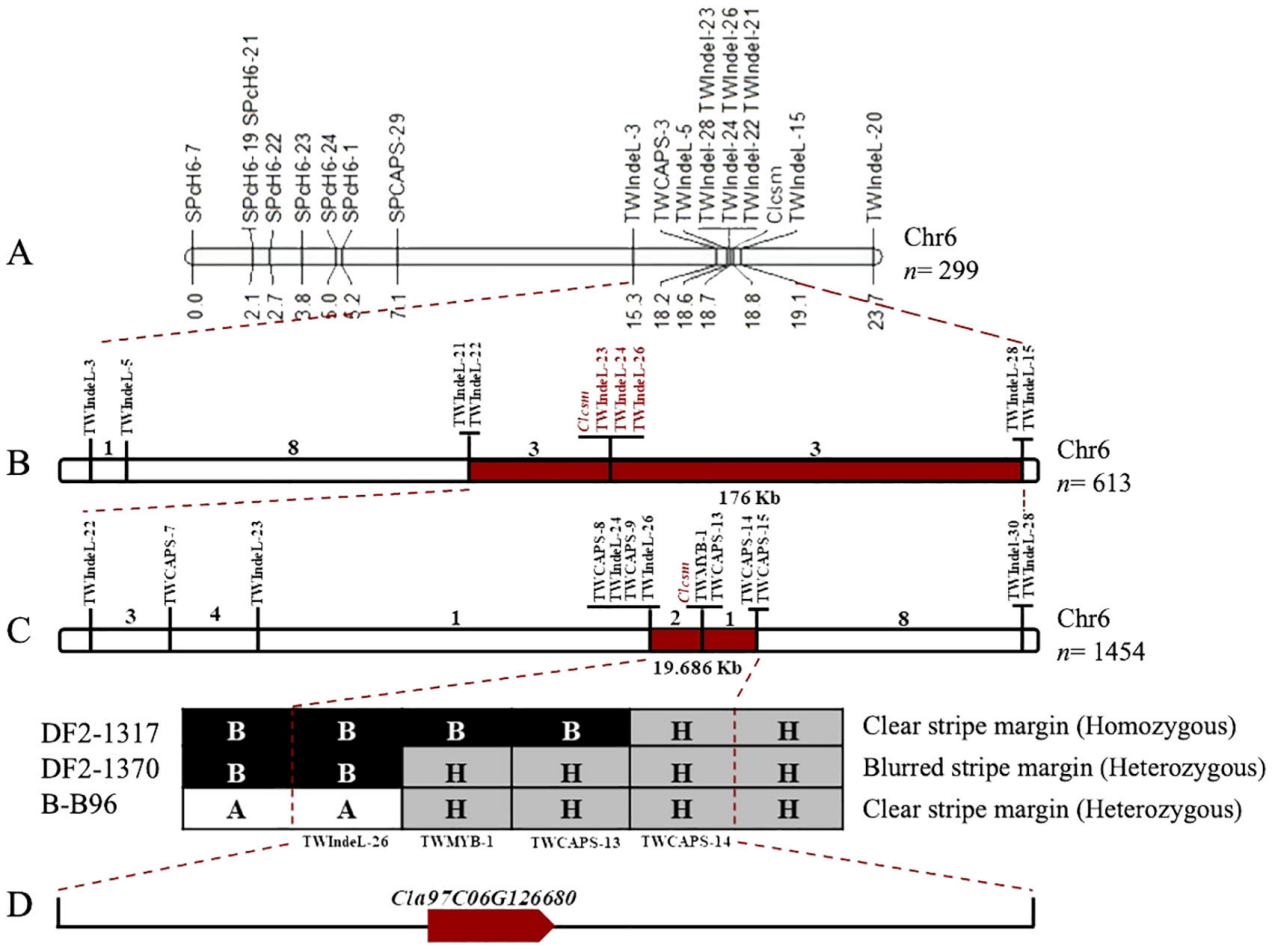
Fine genetic mapping for identification of major *Clcsm* gene in watermelon. **(A)** Primary mapping of *Clcsm* locus using 299 F_2_ individuals. **(B)** Narrowing down of *Clcsm* locus using 613 F_2_ individuals. **(C)** Fine mapping of *Clcsm* locus using extended 1,454 F_2_ individuals. **(D)** A single candidate gene was identified in the fine mapped region.

Fine genetic mapping was performed to verify the major-effect location of the Clcsm locus. Initially, a total of 12 InDels and 7 CAPS polymorphic markers (SPch6-7, SPch6-19, SPch6-21, SPch6-22, SPch6-23, SPch6-24, SPch6-1, SPCAPS-29, TWIndel-3, TWCAPS-3, TWIndel-5, TWIndel-21, TWIndel-22, TWIndel-23, TWIndel-24, TWIndel-26, TWIndel-28, TWIndel-15, and TWIndel-20) were developed within the identified main-effect genetic location (2.40 Mb) on Chr-06 of the watermelon genome (v2). These developed markers were effectively genotyped within the F_2_ segregating population (*n* = 299 individuals), which showed perfect co-segregation across the constructed linkage map. The developed linkage mapping analysis indicated that the Clcsm locus is positioned between two flanking InDel markers (TWIndel-21 and TWIndel-15), and these corresponding markers indicated ~190 kb of genetic interval (**Figure 3A**). Then, a large mapping population (*n* = 613) Clcsm locus is located in the delimited genetic interval of ~176 kb (Chr6:28322846-28499700) between the two closest InDel markers (TWIndel-22 and TWIndel-28), and 13 recombinants were screened (**Figure 3B**).

To further narrow down this target region, an extended F_2_ segregating population (*n* = 1,454 individuals) was utilized within the flanking markers (TWIndel-22 and TWIndel-28). For this purpose, a total of 13 genetic markers (TWIndel-22, TWCAPS-7, TWIndel-23, TWCAPS-8, TWIndel-23, TWCAPS-8, TWIndel-24, TWCAPS-9, TWIndel-26, TWCAPS-13, TWCAPS-14, TWCAPS-15, and TWIndel-30) were genotyped, which indicated that *Clcsm* gene locus exists in a 19.686-kb (Chr6:28428900-28448586) region between two flanking markers (TWIndel-26 and TWCAPS-14), and 21 new recombinants were identified. Finally, two CAPS markers and one InDel marker revealed the recombinants with a homozygous clear peel stripe margin, a heterozygous blurred peel stripe margin, and a heterozygous clear peel stripe margin pattern, respectively (**Figure 3C**). The gene annotation in the Cucurbitaceae genomics (http://www.cucurbitgenomics.org/JBrowse/) database showed that a single candidate gene (*Cla97C06G126680*, encoding MYB98 family transcription factor protein) was present in the delimited genetic interval of 19.686 kb (**Figure 3D**).

### Analysis of genetic mutation and practical validation of developed functional marker

To further analyze the identified *Clscm* gene, Sanger sequencing was used to compare the *Cla97C06G126680* gene sequence between the two comparative parental lines. A total of 14 mutation sites were observed [2 InDels and 12 non-synonymous type SNPs (nsSNPs)], of which two nsSNPs [Chr6:28438793 (A-T) and Chr6:28438845 (A-C)] were significantly associated with clear stripe margin traits in GWAS analysis of the natural germplasm resources of watermelon (**Supplementary Table S3**).

**Figure 4:**
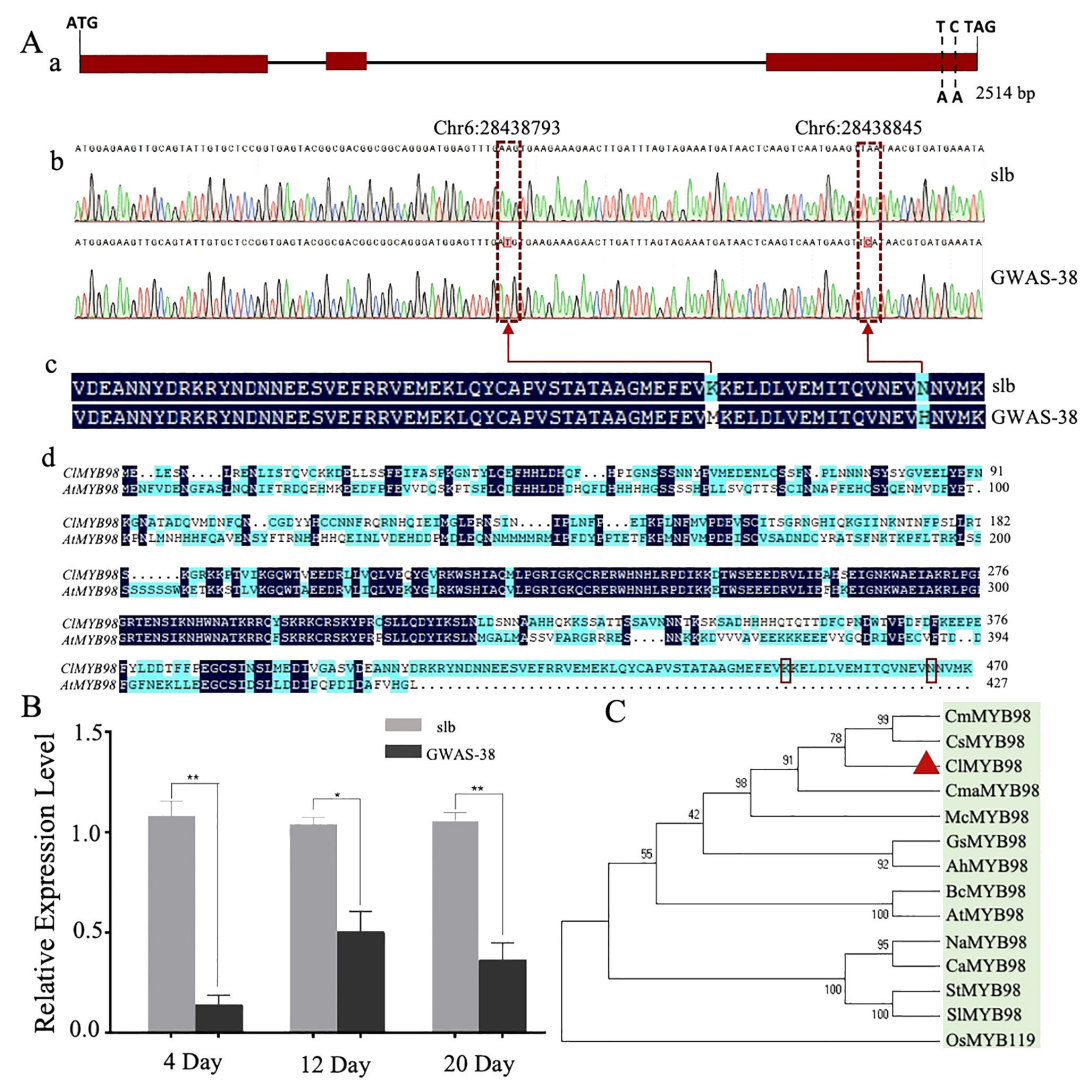
Genetic analysis of major *Clcsm* gene (*Cla97C06G126680*) controlling clear peel stripe margin pattern of watermelon peel. **(A-a)** Structure analysis of *Clcsm* gene; red boxes indicate exons. **(A-b)** Sanger sequencing comparison of candidate genes between two comparative parental lines. **(A-c)** The amino acid sequence difference of *Clcsm* between two parental lines. **(A-d)** The amino acid sequence alignment between proteins of watermelon (ClMYB98) and *Arabidopsis* (AtMYB98); red box lines are candidate mutant amino acids. **(B)** Relative gene expression analysis. **(C)** Phylogenetic associations of Clcsm protein among different species. Asterisks symbols (**, *) are representing the significant results at p < 0.01 and p < 0.05 levels, respectively.

The upstream 2,000-bp sequences of both parents were compared by Sanger sequencing, and 58 mutation sites were found between the parents, which were ubiquitous in the natural germplasm resources of watermelon. Therefore, it is recommended that *Cla97C06G126680* is a candidate gene modulating the clear stripe margin in watermelon (*Clcsm*), while the SNP sites of Chr6:28438845 and Chr6:28438793 are key mutation sites (**Figure 4**). The *Clcsm* gene (encoding the MYB98 family transcription factor protein) exhibited a total length of 2,514 bp and is mainly composed of three exons (**Figure 4A-a**), and two SNP mutation sites [Chr6:28438793 (A-T) and Chr6:28438845 (A-C)] were located on the third exon (**Figure 4A-b**). The amino acid sequence alignment of the candidate gene between two comparative parental lines depicted that the SNP site of Chr6:28438793 (A-T) caused mutations of lysine (K) to methionine (M), and the SNP site of Chr6:28438845 (A-C) triggered mutations of aspartic acid (N) to histidine (H) (**Figure 4A-c**); however, the *Clcsm* gene exhibited a total of 470 amino acid mutation in the comparative sequence analysis of watermelon and *Arabidopsis* (**Figure 4A-d**).

In order to further screen the efficient clear stripe margin cultivars for improving breeding efficiency, the new functional genetic marker (TWMYB-1) was developed based on the mutation site (Chr6:28438845) of the third exon of the *Clcsm* gene. The genotyping of the newly developed marker (TWMYB-1) was performed in the F_2_ mapping population and natural population of GWAS accessions of watermelon, and the stripe margin pattern phenotype was identified according to the digestion products (**Supplementary Figure S1**). When the length of the product after *Mse*l restriction enzyme treatment was 274 bp, it indicated that the sample was a homozygous blurred stripe watermelon strain, and when the length of the digestion product was 232 bp, the sample was a homozygous clear stripe watermelon strain, and when the length of the two digestion products was 274 and 232 bp, it indicated that the sample was a heterozygous clear stripe margin watermelon strain. Overall, the practical validation of the TWMYB-1 marker validated heterozygous lines with blurred stripe margins and homozygous lines with clear stripe margins in segregated F_2_ mapping populations and natural GWAS populations. Thus, it can be deduced that the functional marker (TWMYB-1) is closely linked with the clear stripe margin pattern trait of watermelon peel.

### Analysis of *Clcsm* gene expression pattern and phylogenetic associations

Further, the expression patterns of the candidate *Clcsm* gene (*Cla97C06G126680*) were detected in fruit peel stripe samples of both contrasted parental lines (slb and GWAS-38) at three different developmental stages (4, 12, and 20 DAP) using qRT-PCR analysis (**Figure 4B**). The gene expression levels were noticed to be higher in the fruit peel stripe margin of the slb line (P_1_) than those of the GWAS-38 line (P_2_) at all developmental stages, indicating that the obvious formation of the clear stripe margin pattern started during the earlier growth phase of fruit peel.

Further, the pairwise alignment of the protein sequences was retrieved from the UniProt database (https://www.uniprot.org/), and the phylogenetic association was analyzed using the 13 different plant species (**Figure 4C**). The constructed phylogenesis depicted the significant and higher homologous identity between the five Cucurbitaceae crops [watermelon (ClMYB98), cucumber (CsMYB98), melon (CmMYB98), zucchini (CmaMYB98), and bitter melon (McMYB98)], which were divided into a subgroup; however, the homologous gene sequences of the four solanaceous crops [tobacco (NaMYB98), pepper (CaMYB98), potato (StMYB98), and eggplant (SlMYB98)] were divided into another subgroup, and the rest of the crops basically followed the known phylogenetic relationship between species. However, the highest similarity percentage was observed in the homologous protein sequences of watermelon (ClMYB98) and cucumber (CsMYB), which shared the same clade and exhibited strong relationships, suggesting identical genetic functions. Further, the pairwise structural and functional analysis of protein sequences revealed that Clcsm protein has a higher sequence identity with its homologs, along with non-synonymous type amino acid mutants and the PLN03091 domain of the superfamily Cl33633 (**Supplementary Figure S2**).

### Analysis of candidate DEGs and transcriptional factors

**Figure 5:**
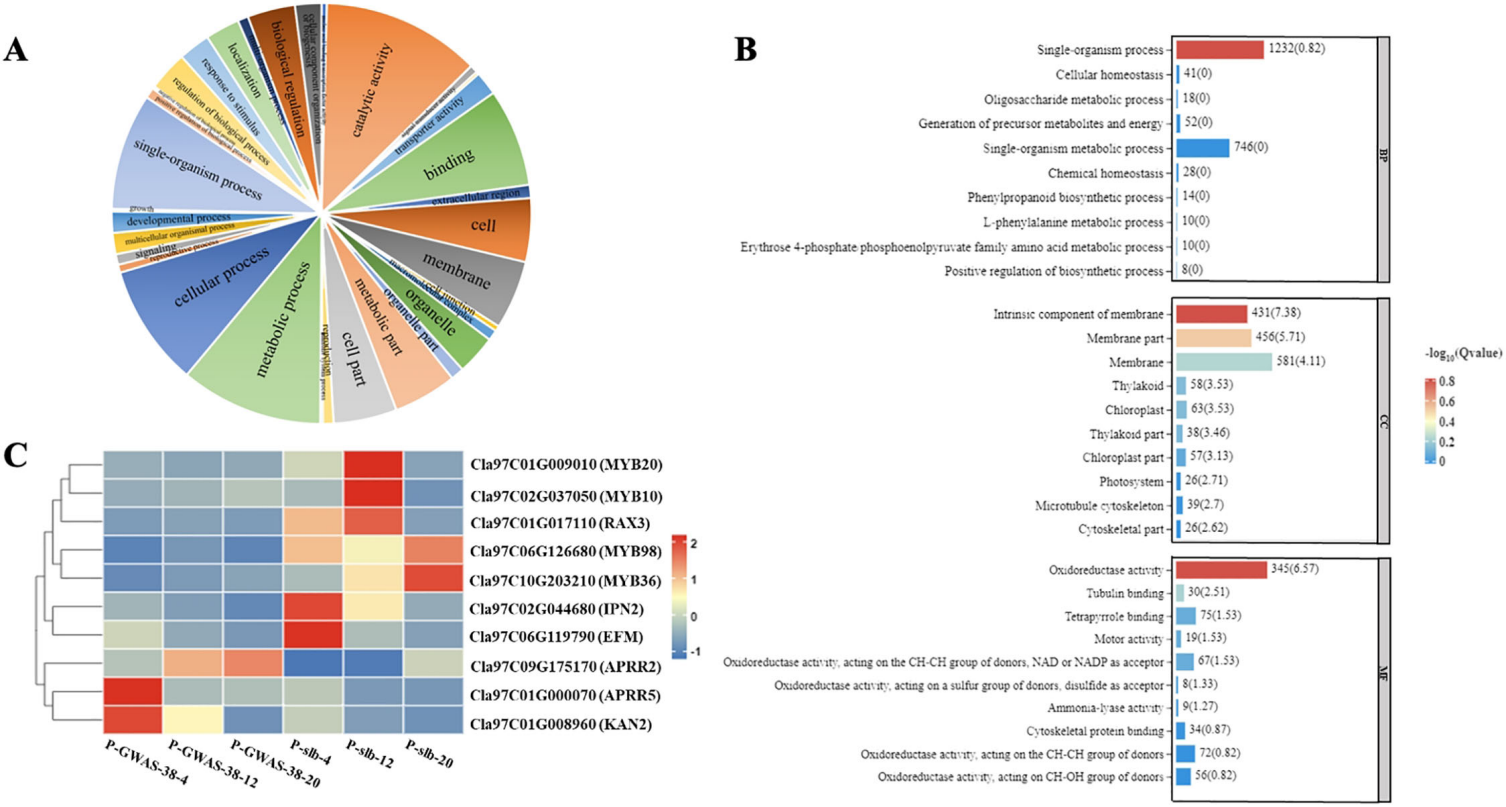
Analysis of DEGs related to ARR-B transcription family involved in differentiated peel stripe margin of watermelon. **(A)** GO functional classification of DEGs. **(B)** GO enrichment analysis of DEGs. **(C)** Heat map of candidate DEGs at three developmental periods of comparative groups. DEGs, differentially expressed genes; GO, gene ontology.

To reveal the gene regulatory mechanism and transcriptional factors involved in the fruit peel stripe margin formation, a total of 18 RNA-Seq (transcriptome) datasets of peel stripes of both parental lines (GWAS-38 and slb) were analyzed at three different developmental periods (4, 12, and 20 DAP). The candidate DEG sets were screened in three randomized groups (G1, G2, and G3, including three comparative subgroups of each) between the same and different genetic materials of contrasted parental lines (**Figure 5**, **Supplementary Figure S3**; **Supplementary Table S4**).

Regarding the G1 group of different parent materials (GWAS-38 and slb) comparison between the same developmental periods, a total of 1,898 DEGs were screened out from the G1-1 subgroup (A38-4 vs. Bslb-4), of which 1,255 genes were upregulated and 643 genes were downregulated. A total of 3,368 DEGs were screened in the G1-2 subgroup (C38-12 vs. Dslb-12), of which 1,631 genes were upregulated and 1,737 genes were downregulated. A total of 2,835 DEGs were screened in the G1-3 subgroup (E38-20 vs. Fslb-20), of which 1,298 genes were upregulated and 1,537 genes were downregulated. However, a total of 204 genes were co-upregulated and 110 genes were co-downregulated (**Supplementary Table S4**; **Supplementary Figure S3**).

Regarding the G2 group of the same genetic material (GWAS-38 line) comparison between different randomized developmental periods, a total of 3,465 DEGs were screened out from the G2-1 subgroup (A38-4 vs. C38-12), of which a total of 1,513 genes were upregulated and 1,952 were downregulated. In the G2-2 subgroup (A38-4 vs. E38-20), a total of 4,833 DEGs were screened, of which 1,972 were upregulated and 2,861 were downregulated. In the G2-3 subgroup (C38-12 vs. E38-20), a total of 1,070 DEGs were screened, of which 341 were upregulated genes and 729 were downregulated genes. However, a total of 68 genes were co-upregulated and 190 genes were co-downregulated (**Supplementary Table S4**; **Supplementary Figure S3**).

Regarding the G3 group of the same genetic material (slb line) comparison between different randomized developmental periods, a total of 1,813 DEGs were screened out from the G3-1 subgroup (Bslb-4 vs. Dslb-12), of which 482 genes showed upregulated expression and 1,331 DEGs had downregulated expression. In the G3-2 subgroup (Bslb-4 vs. Fslb-20), a total of 3,288 DEGs were screened, of which 729 genes showed upregulated expression and 2,559 genes showed downregulated expression. In the G3-3 subgroup (Dslb-12 vs. Fslb-20), a total of 2,150 DEGs were screened, of which 818 genes showed an upregulation trend and 2,332 genes showed a downregulation trend. However, a total of 32 genes showed a co-upregulated expression trend, and 153 genes showed a co-downregulated expression trend (**Supplementary Table S4**; **Supplementary Figure S3**).

In addition, RNA-Seq data revealed a total of 44 transcription factor families involved in the formation of the peel stripe margin pattern of watermelon, among which candidate genes belonging to the ARR-B transcription factor family exhibited a major contribution. The gene structure of the ARR-B transcription family, with the largest set of DEGs, was functionally classified by GO enrichment analysis, and the maximum amount of DEGs was found to be associated with three types of processes (catalytic activity, metabolic process, and cellular process), with the most significant enrichment of DEGs between parents (**Figure 5A**). A total of three molecular functions (single organism process, intrinsic component of membrane, and oxidoreductase activity) may be involved in the regulation of the clear or blurred peel stripe margin (**Figure 5B**). Further, the expression analysis revealed that multiple genes [*Cla97C01G009010* (MYB20), *Cla97C02G037050* (MYB10), *Cla97C017110* (RAX3), *Cla97C06G126680* (MYB98), *Cla97C10G203210* (MYB36), *Cla97C02G044680* (IPN2), *Cla97C06G119790* (EFM), *Cla97C09G175170* (APRR2), *Cla97C01G000070* (APRR5), and *Cla97C01G008960* (KAN2)] in the ARR-B transcription family are involved, exhibiting significant differences between the fruit peel stripe margins of both parents in different comparative and same groups (G1, G2, and G3) of three developmental stages (**Figure 5C**).

### Candidate gene validation using qRT-PCR

**Figure 6:**
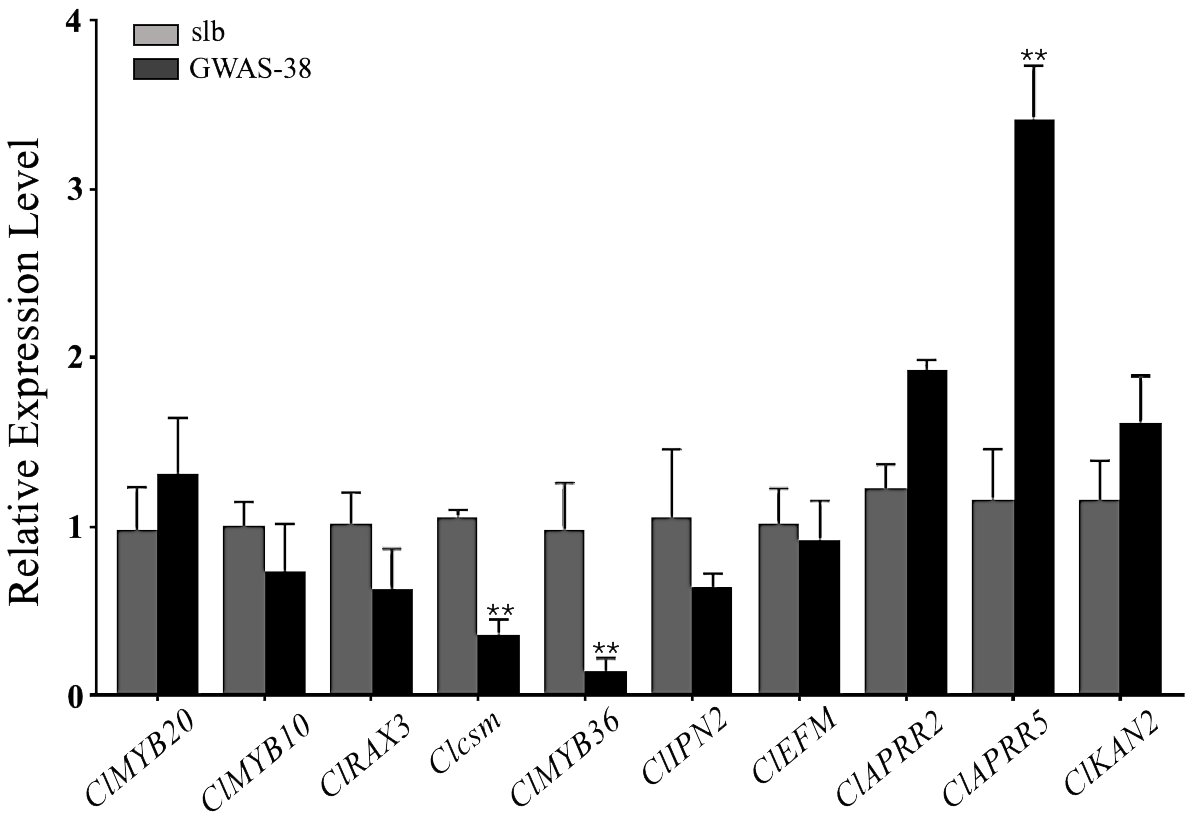
Relative expression level of candidate mapped gene and differentially expressed genes in fruit peel stripe margin of comparative parental lines at 20 days after pollination (DAP). Asterisk symbol (**) is representing the significant results at p < 0.01 level.

The *Clcsm* gene and candidate DEGs (*ClMYB36*, *ClAPRR5*, *ClMYB20*, *ClMYB10*, *ClRAX3*, *ClIPN2*, *ClEFM*, *ClAPRR2*, and *ClKAN2*) were validated in the fruit peel stripe tissues collected on 20 DAP using qRT-PCR analysis (**Figure 6**). The relative expression trends of the *Clcsm* gene (*Cla97C06G126680*) and *ClMYB36* gene (*Cla97C10G203210*) were highly significant in the clear stripe margin pattern line (slb, P_1_) than the blurred stripe margin pattern line (GWAS-38, P_2_); the *ClAPRR5* gene (*Cla97C01G000070*) showed highly significant expression in the blurred stripe margin pattern line (GWAS-38, P_2_); the other seven genes (*ClMYB20*, *ClMYB10*, *ClRAX3*, *ClIPN2*, *ClEFM*, *ClAPRR2*, and *ClKAN2*) did not show any significant differences in expression level between the comparative parental lines. Therefore, it was strongly inferred that the *Clcsm*, *ClMYB36*, and *ClAPRR5* genes may be involved in the formation of clear peel stripe margin patterns; however, the *ClMYB20*, *ClMYB10*, *ClRAX3*, *ClIPN2*, *ClEFM*, *ClAPRR2*, and *ClKAN2* genes may not be involved in the formation of clear or blurred peel stripe margin patterns.

## Discussion

Among the cucurbit crops, few molecular studies on the identification of key genes modulating the peel stripe margin pattern have been conducted in cucumber and melon; however, the mechanism involved in the peel stripe margin pattern has not been well studied in watermelon. Therefore, the study of gene regulatory mechanisms in the peel stripe margin has attracted significant attention in recent years.

Regarding the cucumber (*Cucumis sativus* L.), different types of rind stripe patterns (netting, mottled, and irregular types) are present in various cultivars. The formation of a dense netting pattern over the surface of mature cucumber fruit is supposed to be modulated by one dominant *H* gene (*Csa5G591790*), located in the genetic segment of Chr-05 (~297.70-kb interval) (Wang et al., 2014). The irregular peel stripe pattern was identified to be regulated by a single recessive gene (*Csa1G005490*), located at a narrowed locus of ~144-kb interval on Chr-01 (Song et al., 2019). In melon (*Cucumis melo* L.), three peel patterns (netting, mottled, and striped types) have been proposed, and two major genes (*mt* and *mt-2*) are known to have a dominant effect over the smooth peel surface pattern (Périn et al., 1999; Liu, 2004). The netted peel exhibited multifaceted patterns over the melon fruit surface and was presumed to be modulated through the dominant *Rn* (*N*) gene (Herman et al., 2008). Two genes (*st* and *st-2*) regulating the striped-peel pattern are known as recessive to the non-striped or uniform surface pattern over melon fruit (Danin-Poleg et al., 2002; Liu, 2004; Pitrat, 2006). It was stated that a single dominant gene regulates the major dominance of the high-density pattern compared to the low-density pattern (Yang et al., 2017). BSA-Seq exposed that the main-effect locus on Chr-02 (~280 kb) harboring the major gene (*CmSp-1*) is affecting the dominance of the mottled-peel pattern over the non-mottled peel in melon (Lv et al., 2018). BSA-Seq identified that a dominant gene (*Cmst3*) controlling the striped-peel pattern is located in the delimited region (172.8 kb) on Chr-04 of melon (Liu et al., 2019). Fine mapping revealed a genetic interval of 40.6 kb harboring the candidate *MELO3C026282* gene affecting mottled rind pattern in melon (Shen et al., 2021). BSA-Seq-based genetic mapping showed that two potential genomic regions on Chr-04 (from 0.00 to 2.97 Mb) and Chr-05 (from 0.00 to 2.34 Mb) regulate the formation of the green-spotted fruit rind in wild-type melon. The targeted region predicted that the four genes (*MELO3C003316*, *MELO3C003375*, *MELO3C003388*, and *MELO3C014660*) regulating chloroplast development or chlorophyll biosynthesis may be the best candidate genes, but the functional analysis has not yet been reported (Zhou et al., 2024). In watermelon (*C. lanatus* L.), genetic regulation of striped-peel appearance has been investigated in a few cultivars, and multiple genes underlying main-affect loci have been known to regulate the differential striped types, e.g., the recessive *m* gene for mottled type (Weetman, 1937), the *Yb* gene and *ins* gene for differentiated stripe pattern (Gusmini and Wehner, 2006), the *S* gene for the dominant striped pattern (Yang et al., 2015), and the recessive *csm* gene for the clear stripe margin (Lou and Wehner, 2016). The *ClSP* locus harboring the *Cla97C06G126560* gene [encoding the polygalacturonase-1 non-catalytic subunit beta (*ClGP1*)] was identified as the candidate gene for modulating the striped pattern of watermelon (Liang et al., 2022). A genetic marker (wsbin6-11) was found to be closely linked to the clear peel stripe margin pattern (Kim et al., 2015; Jin et al., 2017). Recently, the main-effect QTL (FRS4.1) affecting the differentiation of the watermelon peel stripe pattern was identified between two flanking CAPS markers on Chr-04 [W4-4Hd (2,031,314 bp) and W4-5Hd (2,731,617 bp)] (Amanullah et al., 2023).

In the current study, we used two contrasted parental lines (with differentiated fruit peel stripe margin patterns) and developed a total of 299 F_2_ mapping individuals. The primary genetic mapping through BSA-Seq exposed that the major-effect genetic locus controlling clear peel stipe margin (*Clcsm*) is localized on Chr-06 of the watermelon genome (97103, v2) (**Figures 2A, B**). This identified result is in line with the previously reported studies where multiple segregating mapping populations have been used and similarly revealed that the fruit peel stripe-associated gene locus is mainly localized on the genetic region of Chr-06 (Gama et al., 2015; Park et al., 2016; Guo et al., 2019; Wu et al., 2019; Yue et al., 2021; Wang et al., 2022, 2023). During data collection in our field trial, we observed that the clear peel stripe margin type parental line was highly correlated with the number of stripes and was negatively correlated with stripe width. Most of the fruits with clear peel stripes in the F_2_ population and the natural GWAS population of watermelon have similar phenotypes to the slb line (clear peel stripe). Fruits with blurred peel stripes in the F_2_ population and natural GWAS population have similar phenotypes to the GWAS-38 line (blurred peel stripe) but have different stripe widths and stripe colors. Further, we used two large F_2_ mapping populations (*n* = 613 and *n* = 1,454), and fine genetic mapping showed that the clear peel stripe margin pattern was modulated by a recessive gene, and this result is consistent with previous studies (Kim et al., 2015; Lou and Wehner, 2016; Jin et al., 2017). In our study, a single *Clcsm* gene (*Cla97C06G126680*, encoding a MYB transcription family protein) was located in the 19.686-kb (Ch6:28428900–28449586) region of Chr-06 (**Figure 3**). Although our identified results are in line with the above-mentioned studies, the chromosomal segments and identified genes are somewhat different, which significantly supports the novel results of our current study. Therefore, we hypothesized that the identified contrasted results may be due to the divergences in the genetic backgrounds of the parent materials used in the existing experiment and previously reported studies, as well as the improved reference genome.

Further, we compared the *Cla97C06G126680* gene sequences between the comparative parental lines through Sanger sequencing and found that there were two non-synonymous type SNPs [Chr6:28438793 (A-T) and Chr6:28438845 (A-C)] in the CDS region, and these SNPs triggered significant variations in amino acids (**Figure 4**), which were significantly related to differentiating the clear and blurred phenotypes of peel stripe margin over the fruits. For efficient marker-assisted selection breeding, the functional molecular marker (TWMYB-1) was developed between the flanking markers of the 19.686-kb region and verified by F_2_ populations and natural germplasm resources (GWAS accession) to assist in the selection of watermelon lines with a clear stripe margin (**Supplementary Figure S1**). At the same time, we used different genetic groups of watermelon with the presence or absence of peel stripe margin (Hou et al., 2022; Liang et al., 2022), stripe width (Lou and Wehner, 2016), and clear/blurred stripe margin (Kim et al., 2015), and we studied the other stripe-related genes. On Chr-06, we discovered an interesting phenomenon of a collinear relationship between melon and cucumber stripes and speculated that there is some relationship between the formations of peel stripe margins in different crops, as reported in our previous study (Gao et al., 2021). Hence, we tried to validate this theory and used the previously developed marker (wsbin6-11), which has been reported to be tightly linked to the clear or blurred margin of peel stripes (Kim et al., 2015). We used the core germplasm (GWAS accessions) and genetic materials preserved in our laboratory and checked the accuracy of this marker. We found no polymorphism in the genetic materials of our laboratory. However, the practical validation of our developed markers (TWMYB-1) in 299 F_2_ mapping populations and natural populations validated the heterozygous lines with blurred stripe margins and homozygous lines with clear stripe margins, respectively (**Supplementary Figure S1**). Thus, we can say that we first reported the *Cla97C06G126680* gene and validated a functional marker for the clear stripe margin of peel stripes. A BLAST comparison with the *Arabidopsis* database revealed that the *AtMYB98* gene has the highest homology with the *Cla97C06G126680* gene (**Supplementary Figure S2**). The phylogenic association and domain architecture analysis of Cla97C06G126680 protein in homologous sequences in other 13 species and the watermelon genome revealed a high percentage of similarity between watermelon (ClMYB98) and cucumber (CsMYB) (**Figure 4**), showing strong relationships and similar genetic functions.

Regarding the candidate DEG identification, we collected differentiated peel samples at the 4, 12, and 20 DAP, and transcriptomic analysis was performed for the detection of highly enriched DEGs (**Supplementary Figure S3**; **Supplementary Table S4**; **Figure 5**). The results showed the candidate *Clcsm* gene belongs to the ARR-B transcription factor family, and the highest number (242) of DEGs was found in this family. The significance GO enrichment analysis was performed on the ARR-B transcription factor family, and the biological processes related to the formation of the clear/blurred peel stripe pattern phenotypes were screened out (**Figure 5A**). A total of three molecular functions were identified (single organism process, intrinsic component of membrane, and oxidoreductase activity) (**Figure 5B**). The significant KEGG enrichment analysis screened out metabolic pathways, mainly including plant–pathogen interactions, phytohormone signal transduction, and circadian rhythm plants. These enriched activities are similarly supposed to be related to the formation of the clear/blurred peel stripe phenotypes of the genetic materials of watermelon used in this study. Further, the expression trend analysis depicted that multiple genes in the ARR-B transcription family exhibited significant differences between the fruit peels of comparative groups (G1, G2, and G3) of three developmental stages (**Figure 5C**).

In a previous study, the cytological observations of fruit peel showed that there is a presence of vascular bundle tissue under the dark surface area of watermelon fruit, and there may be a causal relationship between the vascular bundle and the peel stripe pattern (Korn, 2007; Paris, 2009; Yang et al., 2022). The development of the peel stripe margin is a complex biological process, and multiple biological and metabolic pathways are supposed to be involved in regulating the formation of the peel stripe margin. As far as we know, none of the researchers have conducted in-depth studies so far, and herein, we screened out a total of 10 DEGs and verified them by qRT-PCR analysis, among which the *Clcsm* and *ClMYB36* genes were significant and highly expressed in slb and the *ClAPRR5* gene was highly significantly expressed in the GWAS-38 line (**Figure 6**). In previous studies, it was reported that peel color transition is related to the chlorophyll synthesis pathway, and the expression of most genes required for chlorophyll biosynthesis, e.g., *CHLH*, *HEMA1*, and *GUN4*, is light-dependent and regulated by circadian rhythm (Matsumoto et al., 2004; Enrico and Bernhard, 2009). Among them, the *ClAPRR5* gene belongs to the circadian rhythm substance metabolism pathway. It is speculated that the *ClAPRR5* gene may play a certain role in the formation mechanism of peel stripes by regulating genes related to chlorophyll biosynthesis. The plant–pathogen interaction pathway was found to be highly enriched in DEGs in samples with different peel colors (Chen et al., 2021). In our study, the candidate genes (*Clcsm* and *ClMYB36*) also belong to the plant–pathogen interaction pathway, and it is speculated that these genes have a significant contribution to the formation of the peel stripe margin.

It has been proposed that there is a boundary between cell division and elongation, which is known as the transition boundary, and the boundaries of different cell regions constitute this transition zone (Salvi et al., 2020). It is speculated that there is also a transition zone between the color and stripe area of watermelon peel, and the development trend of this area is the key to determining the differentiated phenotypes of clear and blurred stripe boundaries. Auxin is known as a plant hormone, and the polar transport of this hormone triggers graded auxin development and root transition zone formation (Sabatini et al., 1999; Blilou et al., 2005; Grieneisen et al., 2007). Auxin modulates the supply of transcription factor “*PLETHORA* (*PLT*)”, which similarly exhibits the graded supply (Aida et al., 2004). The higher *PLT* levels in the meristem are essential for maintaining slow cell division, moderate *PLT* levels stimulate rapid cell division, and lower *PLT* levels trigger cell expansion and cause significant differentiation (Galinha et al., 2007; Mähönen et al., 2014). Cytokinin (CK) is another type of key phytohormone responsible for influencing the auxin distribution in the meristem and the *Arabidopsis thaliana response regulator 1* (*ARR1*) transcription factor; however, cytokinin affects the minimum auxin formation in the transition zone (Di Mambro et al., 2017). The genetic regulatory mechanisms required for the formation and stable position of the transition zone were explored, and it was found that the *ARR12*/*PLT* auxin regulatory network contributes to the expansion of the root meristem during initial development, while the *ARR1*/*KRP2/PLT* auxin regulatory network defines the distribution of root meristems. In later developmental stages, *ARR1* activity leads to an increase in *KRP2*, which determines the formation of the actual transition zone and the size of the meristem by inhibiting the formation of cell division (Salvi et al., 2020). In our current study, transcriptional analysis results indicated that the ARR-B transcription family plays an important role in peel stripe formation. It is speculated that ARR-B affects *KRP2*, *PLT*, and auxin network effects through direct or indirect means, thereby controlling the generation and stability of the transition zone and resulting in the appearance of the differentiated phenotype of a clear or blurred peel stripe margin in watermelon.

## Conclusion

The current molecular experiment determined the effective usage of next-generation sequencing (NGS)-based whole-genome BSA-Seq and RNA-Seq for the identification of candidate genes affecting crop traits. Our fine genetic mapping coupled with GWAS analysis signified the main-effect *Clcsm* locus that harbors the *Cla97C06G126680* gene modulating the clear peel stripe margin pattern of watermelon. The allelic diversity, pairwise sequence, phylogenetic association, and qRT-PCR expression in distinct parental lines validated that this major gene is mainly contributing to differentiating the peel stripe margin pattern appearance by causing two nsSNP-based mutations. In addition, RNA-Seq analysis also identified candidate DEGs and ARR-B transcription family factors involved in differentiated peel stripe margin. Finally, two candidate genes (*ClMYB36* and *ClAPRR5*) exhibited significant differentiation in expression profiles for clear and blurred stripe margin patterns. In short, we believe that our research findings would provide new genetic insights and pave the way for understanding the in-depth regulatory mechanisms underlying the formation of peel stripe margin patterns, as well as assist in marker-assisted breeding for the development of superior cultivars.

## Data Availability

The datasets presented in this study can be found in online repositories. The names of the repository/repositories and accession number(s) can be found below: https://www.ncbi.nlm.nih.gov/, PRJNA1070357.
