# Supplementary material for: Fine genetic mapping and transcriptomic analysis revealed major gene modulating the clear stripe margin pattern of watermelon peel
Source: Front Plant Sci. 2024 Sep 4;15:1462141. doi: 10.3389/fpls.2024.1462141 (PMC11409187; doi:10.3389/fpls.2024.1462141)
Supplement: Supplementary file 2 [file Image1.pdf]

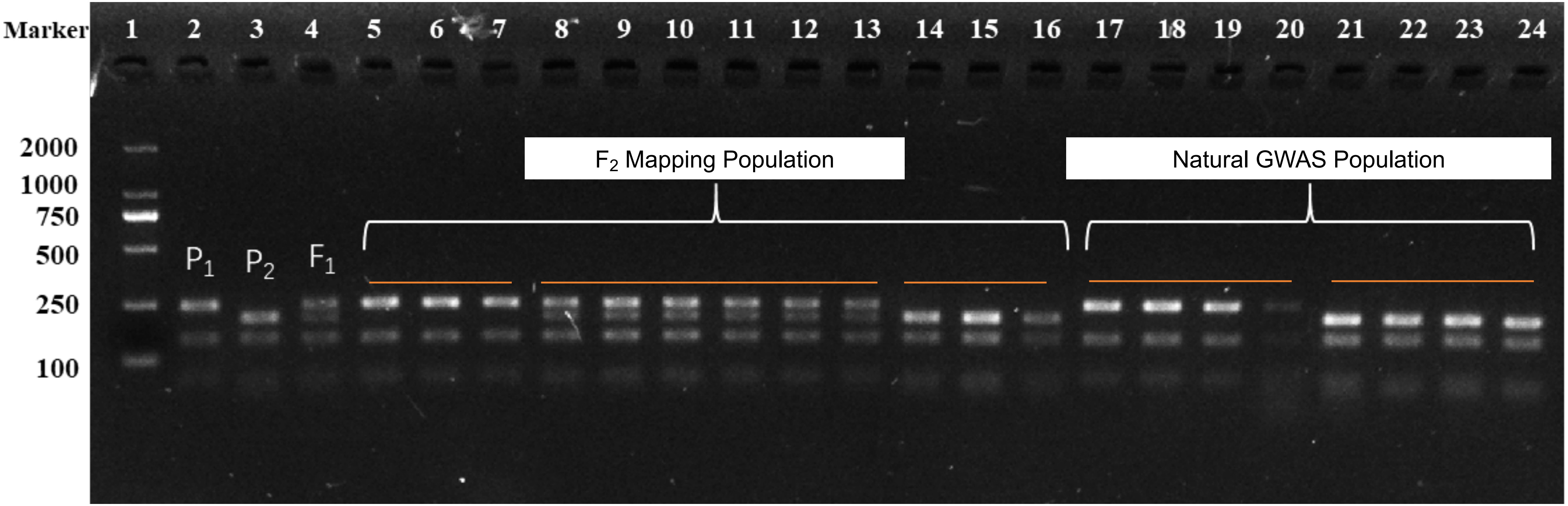

Supplementary Figure S1 | The practical validation of TWMYB-1 marker in selected F_2 and natural GWAS populations of watermelon. Note: Functional molecular marker validation lanes in isolated and natural populations are as followed; 1) DM2000 Marker; lanes 2-4) bi-parents and F_1; lanes 5-7) watermelon homozygous lines with blurred peel stripes margin; 8-13) watermelon heterozygous lines with blurred peel stripes margin; 14-16) watermelon homozygous lines with clear peel stripes margin; 17-20) natural watermelon populations with homozygous lines having blurred peel stripes margin; 21-24) natural watermelon population homozygous lines having clear peel stripes margins, respectively.

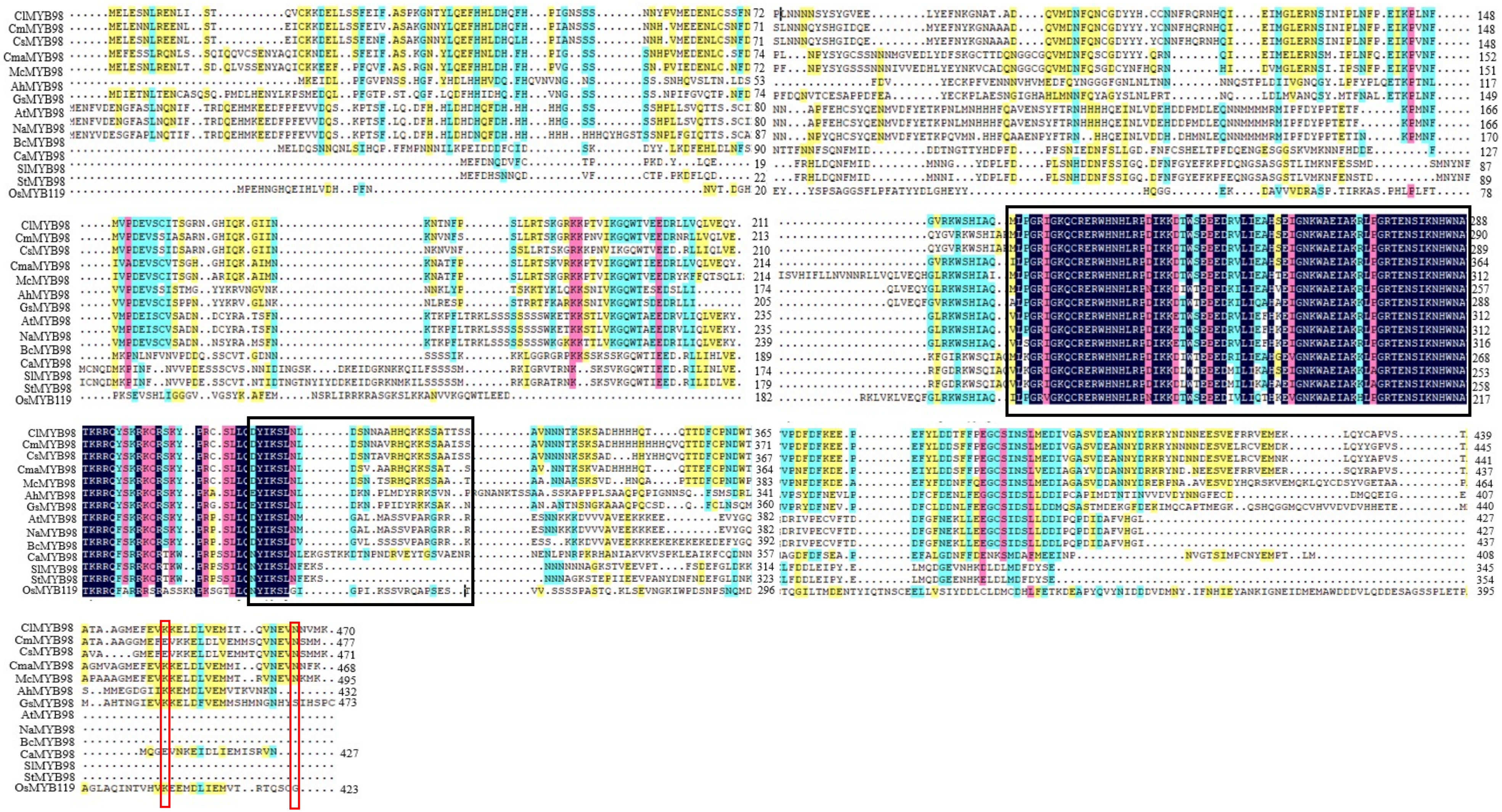

Supplementary Figure S2 | The comparison of protein sequences of MYB98 protein of watermelon with MYB in 13 other species. Note: Black frame line represents PLN03091 domains (MYB), red frame line represents non-synonymous mutant amino acids between parents.

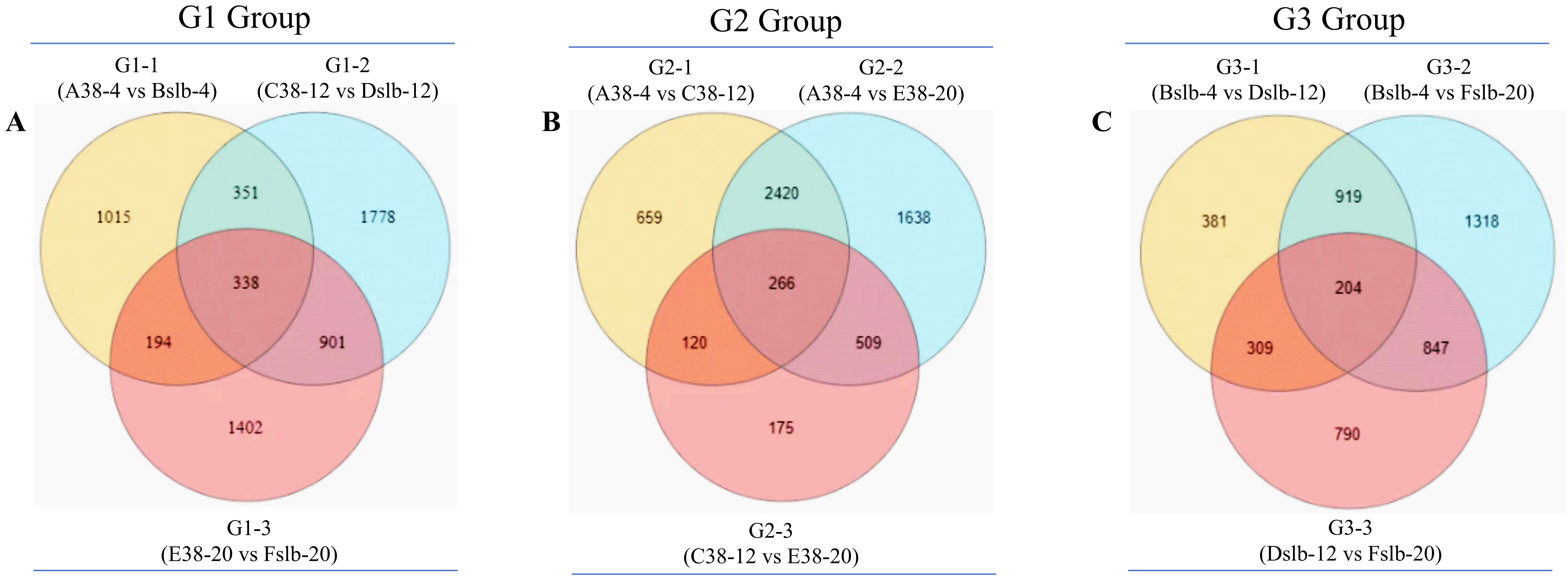

Supplementary Figure S3 | Venn diagram of categorized differentially expressed genes (DEGs) in different groups, including three subgroups of randomized developmental stage of comparative and same genetic materials (two parent lines). (A) Venn diagram of DEGs in G1 group, including subgroups (G1-1, G1-2, G1-3). (B) Venn diagram of DEGs in G2 group, including subgroups (G2-1, G2-2, G2-3). (C) Venn diagram of DEGs in G3 group, including subgroups (G3-1, G3-2, G3-3), respectively.
